# Development of predicitve models to distinguish metals from non-metal toxicants, and individual metal from one another

**DOI:** 10.1186/s12859-020-3525-7

**Published:** 2020-12-03

**Authors:** Zongtao Yu, Yuanyuan Fu, Junmei Ai, Jicai Zhang, Gang Huang, Youping Deng

**Affiliations:** 1 Department of Laboratory Medicine, Affiliated Taihe Hospital of Xi’an Jiaotong University Health Science Center, Shiyan, 442000 Hubei China; 2 Department of Laboratory Medicine, Shiyan Taihe Hospital, College of Biomedical Engineering, Hubei University of Medicine, Shiyan, 442000 Hubei China; 3 Department of Internal Medicine, Rush University Medical Center, Chicago, IL 60612 USA; 4 Bioinformatics Core, Department of Quantitative Health Sciences, University of Hawaii John A. Burns School of Medicine, Honolulu, HI 96813 USA; 5 Shanghai Key Laboratory for Molecular Imaging, Shanghai University of Medicine and Health Sciences, Shanghai, 201318 China

**Keywords:** Biomarker, Microarray, Toxic heavy metals, Classification

## Abstract

**Background:**

Evaluating the toxicity of chemical mixture and their possible mechanism of action is still a challenge for humans and other organisms. Microarray classifier analysis has shown promise in the toxicogenomic area by identifying biomarkers to predict unknown samples. Our study focuses on identifying gene markers with better sensitivity and specificity, building predictive models to distinguish metals from non-metal toxicants, and individual metal from one another, and furthermore helping understand underlying toxic mechanisms.

**Results:**

Based on an independent dataset test, using only 15 gene markers, we were able to distinguish metals from non-metal toxicants with 100% accuracy. Of these, 6 and 9 genes were commonly down- and up-regulated respectively by most of the metals. 8 out of 15 genes belong to membrane protein coding genes. Function well annotated genes in the list include *ADORA2B*, *ARNT*, *S100G*, and *DIO3*. Also, a 10-gene marker list was identified that can discriminate an individual metal from one another with 100% accuracy. We could find a specific gene marker for each metal in the 10-gene marker list. Function well annotated genes in this list include *GSTM2*, *HSD11B*, *AREG*, and *C8B*.

**Conclusions:**

Our findings suggest that using a microarray classifier analysis, not only can we create diagnostic classifiers for predicting an exact metal contaminant from a large scale of contaminant pool with high prediction accuracy, but we can also identify valuable biomarkers to help understand the common and underlying toxic mechanisms induced by metals.

## Background

The last decade has seen a growing interest in the metal toxicity towards environmental contamination and human health, however, efficient and accurate assessment of the potential environmental metallic and non-metallic contamination remains an enduring challenge in environmental health protection [1]. Traditional methods are very time consuming, inefficient, and expensive, so limited number of chemicals can be tested [2, 3]. Applications of new approaches, including massive sequencing techniques make toxic genomics strategies to classify hepatotoxic and non-hepatotoxic compounds and explore these molecular mechanism [4].

The liver is the main organ of metabolism and also the main organ of the toxicity of chemical compounds [5, 6]. The primary culture method of hepatocytes provides an in vitro system that is convenient to do toxic chemicals screening. Cell culture can also reduce the damage to animals, reduce costs, and make research in vivo feasible [7]. The use of in vitro system screening for the treatment of human diseases and new drug research as well as molecular mechanisms has a long history [7]. In this study, gene expression profiles were generated from rat primary hepatocytes treated with 105 different compounds, including 9 heavy metals (Selenium, Chromium, Arsenic, Lead, Cadmium, Nickel, Zinc, Copper, and Tungsten) and their respective vehicle controls [8, 9]. The microarray classifier is analyzed by comparing different feature types, sizes, and two feature selection methods based on LibSVM classification algorithm [10]. Microarray classifiers analyze the prospects in the field of toxic genomics in identifying biomarkers to predict unknown samples and to help understand toxicity mechanisms [8, 11].

## Material and methods

### Chemicals

The chemicals were described in previous study [12].

### Cell culture

The primary rat hepatocytes, rtNHeps (AC-2630), were isolated from male Sprague Dawley, and reconstituted in HCM supplemented with ascorbic acid, fatty acid-free bovine serum albumin, transferrin, insulin, recombinant human epidermal growth factor, hydrocortisone 21 hemisuccinate, Gentamicin sulfate, and Amphotericin B immediately upon receipt. 3 × 106 cells were seeded in Type 1 collagen-coated T-75 flask following by incubation overnight at 37° with 5% CO_2_.

The cells were replenished with fresh HCM and dosed in triplicate flasks with the non-toxic concentration of each compound at 1% DMSO (v/v) or with solution at 1% water (v/v). Every 3 chemicals used a solvent control. A total of 105 chemicals were used, and each chemical has 3 treatments plus controls. Cells were collected after 24 h’ exposure for RNA extraction.

### Total RNA extraction

Total RNA was extracted from about 30 mg cell pellet according to RNeasy kits (Qiagen) manual instruction. The RNA concentrations were measured by NanoDrop ND-1000 Spectrophotometer (NanoDrop technologies, Wilmington, DE, USA). The integrity and quality of total RNA was determined on an Agilent 2100 Bioanalyzer (Palo Alto, CA).

### Microarray hybridization

Rat whole genome oligo arrays in the format of 4X44K were purchased from Agilent (Santa Clara, CA). Sample cRNA synthesis, labeling, hybridization, and microarray processing were performed according to manufacturer’s protocol “One-Color Microarray-Based Gene Expression Analysis” (version 1.0). In brief, the Agilent One-Color Spike Mix (part number 5188–5282) was diluted 5000-fold and 5 μl of solution mixed with 1 μg RNA samples prior to labeling reactions which were performed using the Agilent Low RNA lnput Linear Amplification Kit in the presence of cyanine3-CTP. And then the labeled cRNA was hybridized to individual arrays at 65 °C for 17 h using Agilent’s Gene Expression Hybridization Kit. After washing, the arrays were scanned at PMT levels 350 setting using GenePix 4200AL Scanner (Molecular Device lnc.), the Agilent Feature extraction software (V.9.5.1) was used to automatically find and place microarray grids, reject outlier pixels, accurately determine feature intensities and ratios, flag outlier pixels, and calculate statistical confidences.

### Microarray data analysis

The raw data was processed with GeneSpring version 7.0 and 10.0 (Agilent). The sample quality control was based on the Pearson Correlation. The sample was excluded when its average correlation with other samples less than 0.8. If the scanned intensity was less than 5.0 for a probe, it was transformed to 5.0. A per chip (within) array normalization was performed using 50 percentile values of all the probe values in the array. Per gene (between) array normalization was conducted using either the median value of a gene across all samples (median based normalization) or relative control samples (control based normalization) in the experiment. Probe features were first filtered by “present” or “absent” flags using the Agilent Feature Extraction 9.5.1 software. Probes were included for further analyses when they present in more than 80% samples of all the arrays. Data were subsequently Log (base 2) transformed for statistical analyses. Initial feature filtering was conducted by One-Way ANOVA unequal variance with two-tail *P* < 0.05.

### Feature selection

Base on the comparation results of different feature selection methods in our previous study [12], Support Vector Machine - Recursive Feature Elimination (SVM-RFE) [13] and InfoGain were used for feature selection. SVM-RFE is an algorithm for feature selection by using SVM in a wrapper-style which is much more robust to data overfitting than other methods [8]. Weka program was used for the rest of methods [14].

### Classification algorithms and error estimation

LibSVM algorithm was performed to do classification. The more details about the different classification algorithms described elsewhere [12]. Ten-fold cross-validation with 10 iterations was conducted to estimate the cross-validation error. The classification and prediction accuracy was also caculated for all the classes and samples.

## Results

### Development of predicitve models for distinguishing metals from non-metal toxicants

In order to build predictive models to distinguish metals from non-metal toxicants, microarray experiements were developed using Agilent Rat Whole Genome Array (4X44k). Cultured primary rat hepatocytes were treated in triplicate with distinctive 105 compounds including 9 metals as well as respective vehicle controls for 24 h, subsequently RNAs were isolated for array hybridization. At least four biological replicates for each compound were used and a total of 531 array samples were generated. The experiments were conducted in2 years. In year 2007, total 168 array samples were produced, and 363 array samples were hybridized in year 2008. For each dataset, all the compounds were included. The 2007 dataset (Dataset 1) contained 12 metal samples and 156 non-metal samples, and the 2008 dataset (Dataset 2) contained 30 metals samples and 333 non-metal samples. To construct reliable models, we have trained on 2007 dataset and built predictive models to distinguish metal samples from non-metal samples in 2008 dataset and vice versa.

**Fig. 1:**
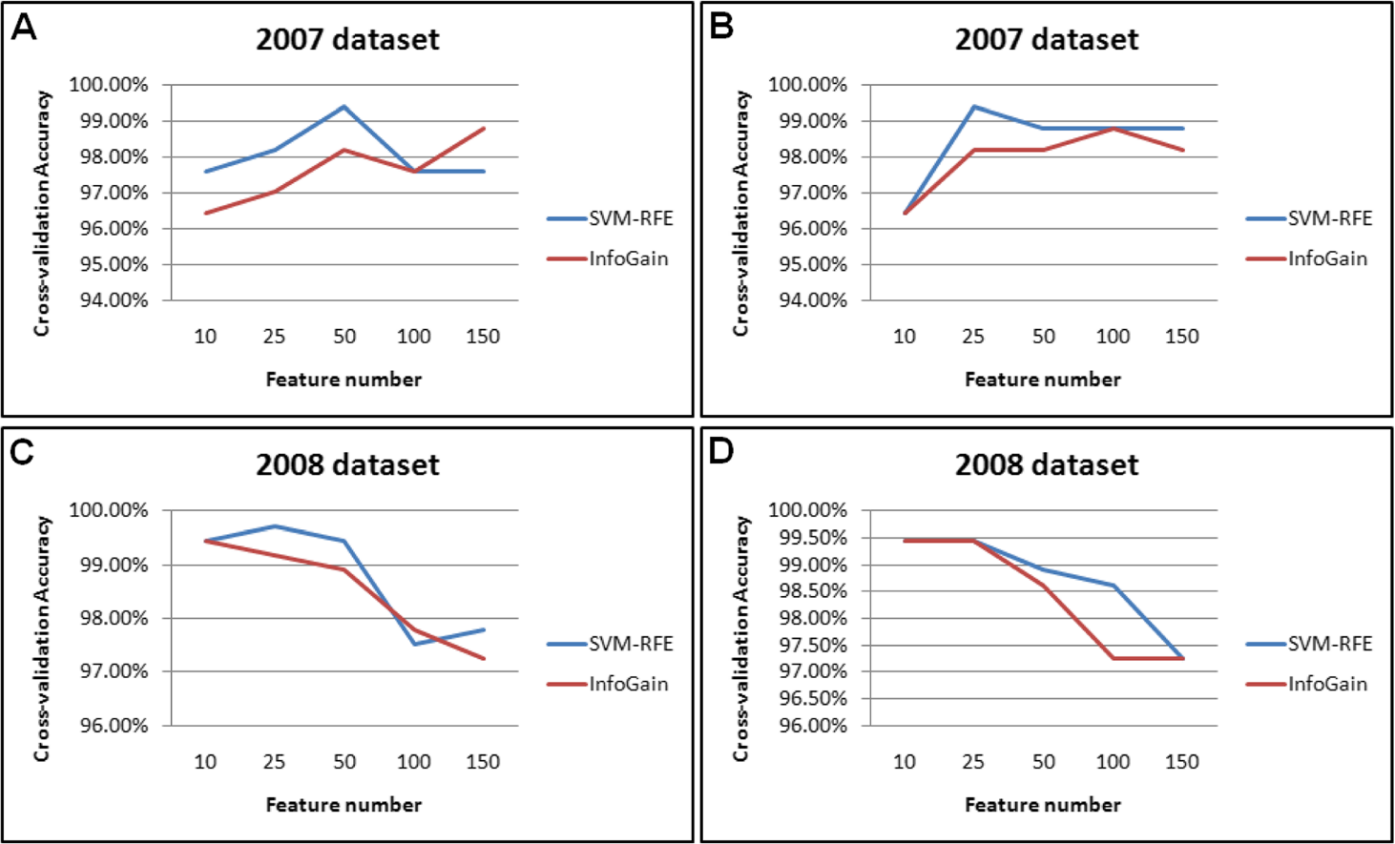
Development of predicitve models for distinguishing metals from non-metal toxicants. **a**. SVM-RFE and InfoGain methods reached their highest accuracy at the feature size 50 and 150 respectively in 2007 dataset (Dataset 1). **b** SVM-RFE and InfoGain methods reached their highest accuracy at the feature size 25 and 100 respectively in 2007 dataset (Dataset 1). **c** SVM-RFE and InfoGain methods reached their highest accuracy at the feature size 25 and 10 respectively in 2008 dataset (Dataset 2). D Both SVM-RFE and InfoGain methods reached their highest accuracy at the feature size 25 in 2008 dataset (Dataset 2)

A total of 25 probe sets (features) were selected for predictive model building. Features were first filtered by comparing metal samples and non-metal samples as well as comparing metal samples and metal related control samples using 1.5 fold change and a T-test with a cutoff *p*-value less than 0.05 for both datasets as well as combined datasets. Only overlapped filtering passing both comparisons were further for model constructions. So, three sets of probes (features) were produced: features based on Dataset 1 (Feature 1), features based on dataset 2 (Featue 2) and features based on combined dataset (Combined features). Feature 1 and 2 were used to train on Dataset1 and Dataset 2 repectively and combined fetures were employed to train both Dataset 1 and Dataset 2 separately. These features were subjected to two further feature selection methods: Support Vector Machine Recursive Feature Selection (SVM-RFE) and InfoGain algorithms. A libSVM classification algrothm was then used to build predict models based on two classes: metal and non-metal classes and 10 fold cross- validation was used to evaluate the model accuracy. As illustrated in Fig. 1, the overall cross validation accuracy for three sets of features at both datasets could reach over 96%. No matter what type of datasets and features were used, the feature selection method SVM-RFE usually achieved higher accuracy than InfoGain method, and this has been validated elsewhere [8, 15, 16]. The feature sizes varied to obtain the highest accuracy depending on the feature types, feature selection methods as well as training datasets. For instance, for training Dataset 1 using Feature 1, SVM-RFE and InfoGain methods reached their highest accuracy at the feature size 50 and 150 respectively (Fig. 1a), whereas the numbers were 25 and 100 for SVM-RFE and InfoGain (Fig. 1b). By comparing the averaged accrucy between two training datasets, the accuracy of Dataset 2 was slightly higher than Dataset 1. By averaged accuracy using Feature 1 or combined features to training the same Dataset 1 or 2 was comparable. The highest accuracies were more than 99.00% for both training datasets using three sets of features, indicating the gene expression profiles possess the protential ability to distinguish metal from non-metal toxciants.

### Prediction of metal from non-metal toxicants using independent datasets

**Fig. 2:**
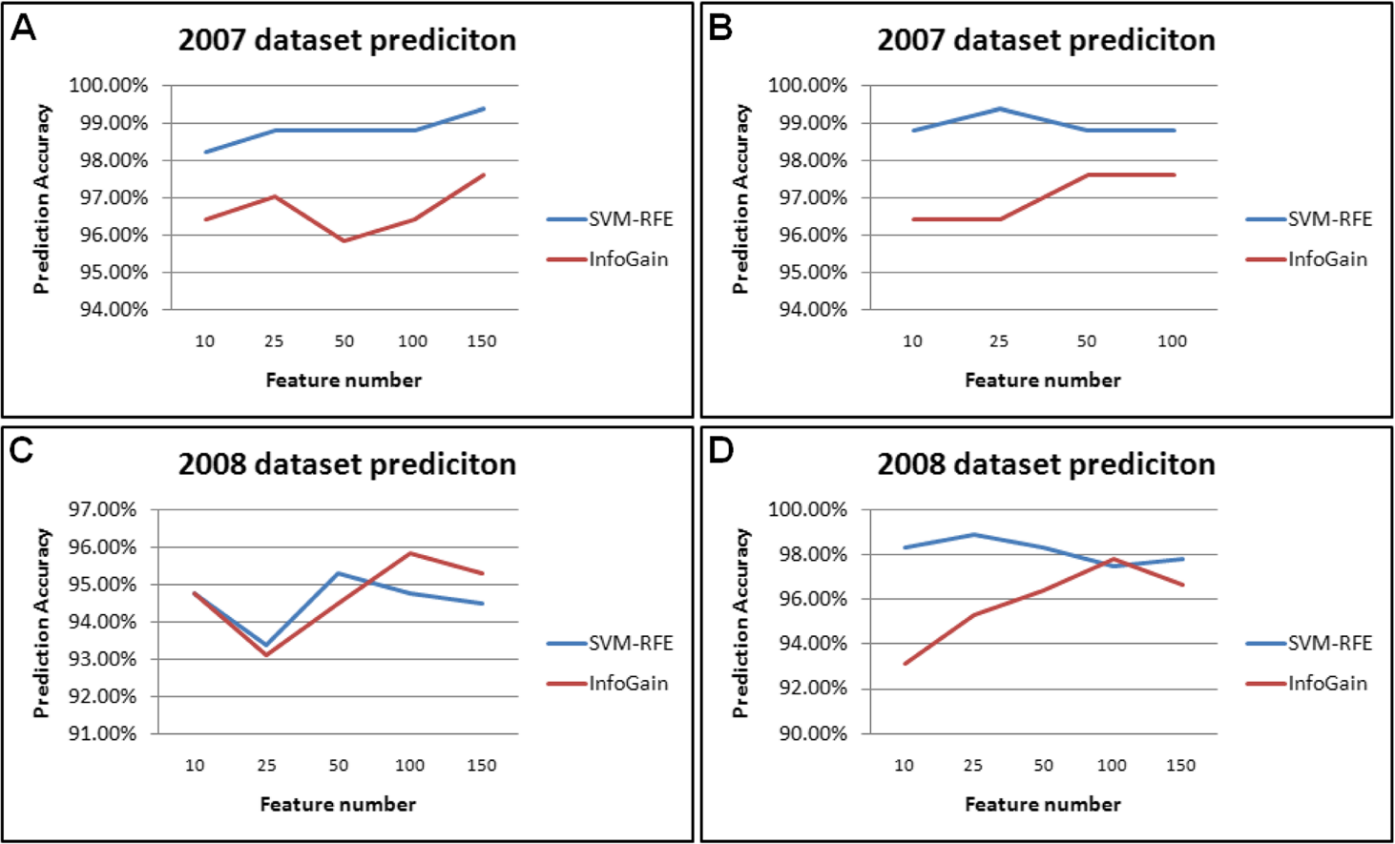
Prediction of metal from non-metal toxicants using independent datasets. **a** SVM-RFE feature selection method was overall better prediction accuracy than InfoGain in 2007 dataset (Dataset 1). **b** A higher prediciton accuracy was observed when using combined features trained model to predict 2007 dataset (Dataset 1). **c** A higher predcition accuracy was observed for predicting 2008 dataset (Dataset 2) using Feature 1. **d** A higher predcition accuracy was observed for predicting 2008 dataset (Dataset 2) using combined feaures

We applied our training models to predict metal samples and non-metal samples. To test the reliability of our models, the training datasets and prediction datasets were different. We used Dataset 1 trained model to predict Dataset 2 or vice versa. Since higher training accuracy might not always result in higher prediction accurcy, we built a series of models to perform the prediction. As shown in Fig. 2, whaterver training models or datasets were used for prediction, the feature selection method SVM-FRE was overall better prediction accuracy than InfoGain, which was consistent with the training accuracy. Using Feature 2 trained Dataset 2 as model to predict Dataset 1, a obviously higher prediciton accuracy was shown in Fig. 2a with an accuracy at least 98.00% for the SVM-RFE feature selection method, and the highest accuracy could reach over 99.00% using 150 features resulted from SVM-RFE. Similarly, we also observed a surprisingly higher prediciton accuracy when using combined features trained model to predict Dataset 1, and the smallest prediction accuracy present in Fig. 2b was 98.81% and the highest accuracy was 99.40% with only one sample miss-classified based on the SVM-RFE feaure selection.

The averaged prediction accuracy using Dataset 1 trained model to predict Dataset 2 was much lower than using Dataset 2 trained model to predict Dataset 1. Nevetheless, we still saw a reasonable high predcition accuracy for predicting Dataset 2 using either Feature 1 (Fig. 2c) or combined features (Fig. 2d). The highest accuracy using combined feaures trained Dataset 1 model to predict Dataset 2 could reach over 98.90% (Fig. 2d). Our results suggest that our extablished models can be used to predict indpendent datasets and yield a convincing accuracy.

### The best predictive model and gene signature for discrimination of metal from non-metal toxicants

To search for the best predcitive model using minimum probe sets, we conducted further training and predcitions based on our above comparisons. We only focused on using combined features trained Dataset 2 to predict Dataset 1, because its averaged prediction accuracy was higher than that of trained Dataset 1. Also, using combined features resulted from more samples should be more reliable and better for the future application to predict unknown samples. Since only one sample was misclassified using the 25 probe sets based on combined features trained Dataset 2 to predict dataset1 after SVM-RFE feature selection, we then thought whether we could find less than 25 probe sets for the prediction. As shown in Table 1, the feature number from 14 to 16 showed 0 error for classifing Dataset 2, and at most 1 error for predicting Dataset 1. Meanwhile, a cross validation for the combined dataset was proceeded to see if we could correctly classify the samples in the dataset. Interesingly, using 15 probe sets based on SVM-RFE, the cross validation error for both Dataset 2 and combined dataset were 0, and the prediction error for Dataset 1 was also 0. However, if increasing probe set number to 16 or more, they were not all 0, thus these 15 probe sets could be considered as a efficient signature (Table 1) to descriminate metal from non-metal toxicants.

**Table 1:** The best predictive model and gene signature for discrimination of metal from non-metal toxicants

No. of probe sets	Cross validation error		Prediction error (D2 to D1)	Probe set ID
	D2	C		
1	8	8	9	A_44_P915194(FAM174B)
5	4	5	8	+ A_42_P546708(KHDRBS3), A_44_P1034910(RTN2), A_42_P537091(FAM12B), A_43_P11261(AHNAK2)
7	3	4	5	+ A_44_P593735(TC632928), A_42_P829301(SLC1A5)
8	3	3	4	+ A_44_P427814(IGH-6)
10	2	3	2	+ A_42_P537051(FAM70B), A_44_P1005988(CDIG2)
14	0	1	1	+ A_43_P11561(ARNT), A_44_P1011716(ADORA2B), A_43_P11444(S100G), A_44_P608892(TC596871)
15	0	0	0	+ A_43_P11861(DIO3)
16	0	1	1	+ A_44_P175654
18	2	1	1	+ A_44_P1040207, A_44_P426107
25	2	2	1	+ A_43_P21000, A_44_P299835,A_44_P1040926 A_44_P751206 A_44_P961496 A_44_P471440 A_43_P21816

### Gene expression pattern and functional analysis of the gene signature that discriminates metal from non-metal toxicants

We then tried to understand the underlying mechanism of how these 15 probe sets (genes) could distinguish metal from non-metal toxicants. To achieve this goal, we performed a two-way hierarchical analysis using averaged metal and metal specific control samples based on the 15 genes.

**Fig. 3:**
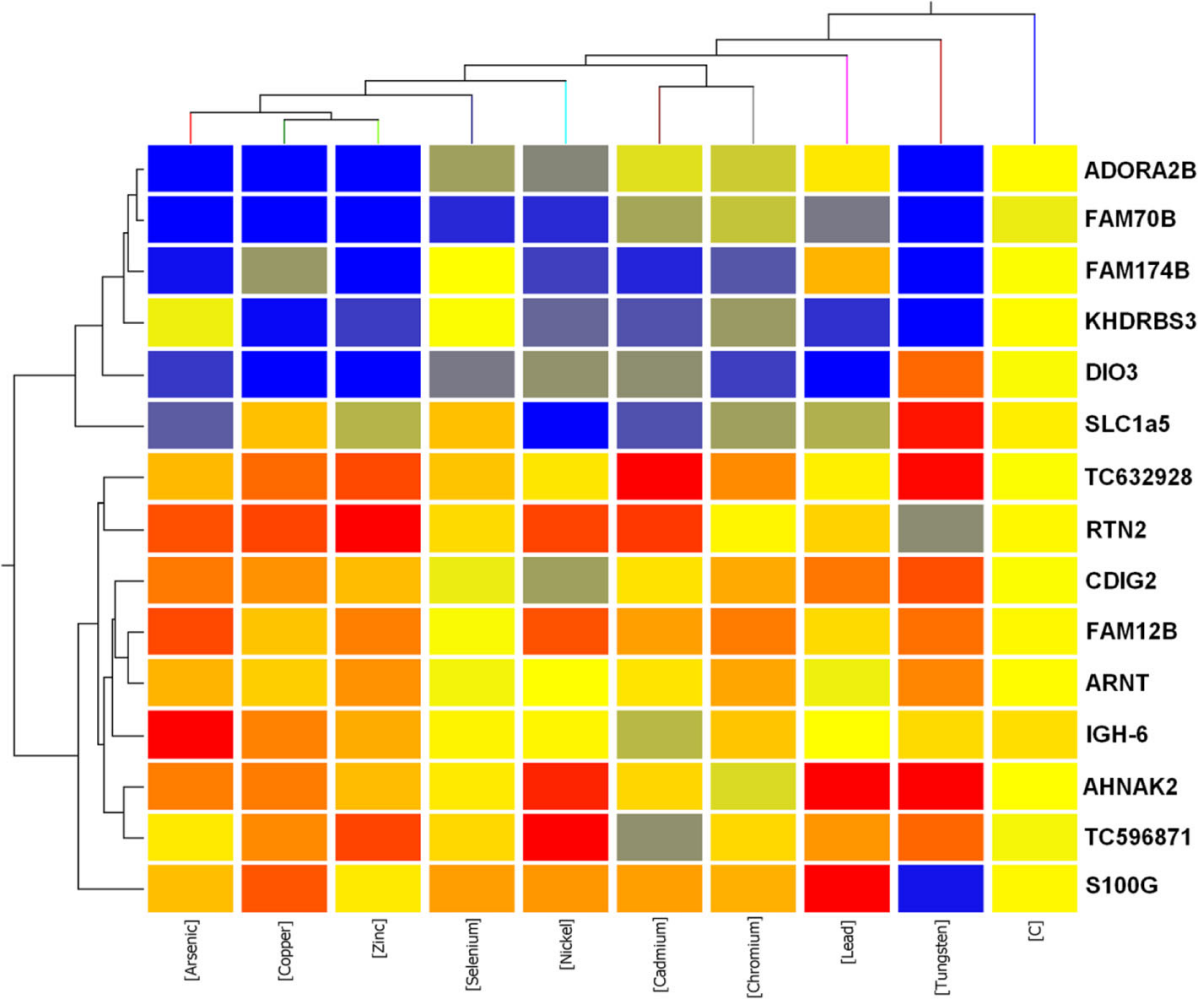
Heatmap shows the gene expression pattern and functional analysis of the gene signature that discriminates metal from non-metal toxicants

From vertically view, as what we expected, the dendrogram (Fig. 3) divided into two big clusters: the control formed one cluster and all the metals formed the other cluster. In the metal cluster, Tungsten alone was in a separated group and all the other metals were in another group. Arsenic, Copper and Zinc fell into a subgroup, indicating that these three metals have more similar gene expression patterns. Cadmium and Chromium clustered together to form a subgroup (Fig. 3).

From horizontally view, the genes were clearly clustered into two groups (Fig. 3). Six genes formed one cluster (Cluster 1), including adenosine A2b receptor (*ADORA2B*), family with sequence similarity 70, member B (*FAM70B*), family with sequence similarity 174, member B (*FAM174B*), KH domain containing, RNA binding, signal transduction associated 3 (*KHDRBS3*), Type 3 iodothyronine deiodinase (*DIO3*) and solute carrier family 1 (neutral amino acid transporter) member 5 (*SLC1A5*). The other 9 genes formed another cluster (Cluster 2), including TC632928, reticulon 2 (*RTN2*), cadmium-inducible gene (*CDIG2*), family with sequence similarity 12, member B (epididymal) (*FAM12B*), aryl hydrocarbon receptor nuclear translocator (*ARNT*), immunoglobulin heavy chain 6 (heavy chain of IgM) (*IGH-6*), AHNAK nucleoprotein 2 (*AHNAK2*), TC596871 and S100 calcium binding protein G (*S100G*). Interestingly, the 6 genes in Cluster1 were commonly down-regulated by all the metals versus control. For instance, the gene *FAM70B* was downregulated by all the metals, *ADORA2B* was downregulated by all the metals except Lead, *KHDRBS3* was almost repressed by all the metals except Selenium, and *DIO3* was downregulated by all the metals except it was induced by Tungsten. In contrast, the 9 genes in Cluster 2 were largerly upregulated by all the metals. For example, the gene *TC632928* was upregulated by all the metals. *RTN2* and *SG100G* whose expression was elevated by all the metals except they were repressed by Tungsten. *AHNAK2* was induced by all the metals except Chromium, and *IGH-6* was alomst upregulated by all the metals except Cadmium. Our results indicated that the metals shared an interesting mechanism through commonly regulating a list of genes, which might explain why the metals could be distinguished from non-metal toxicants. One gene called cadmium-inducible gene (*CDIG2*) was indeed to be induced by Cadmium, but also induced by almost all other metals except Nickel and Selenium. The confirmation of early finding indicated that the microarray data quality is good.

According to Gene Ontology analysis, eight genes including included *ADRA2B*, *DIO3*, *SLC1A5*, *FAM70B*, *FAM174B*, *FAM12B*, *RTN2* and *S100G* out of these 15 markers belong to membrane part. This very high enrichment of membrane proteins encoded genes in the markers, indicating that the metals was distinguished from non-metal toxicants by mainly targeting membrane proteins. Canonical pathway analysis turned out a couple of genes invovled in multiple pathways (Table 2). The upregaulted gene *ARNT* is invovled in Hypoxia Signaling in the Cardiovascular System, Renal Cell Carcinoma Signaling, VEGF Signaling, HIF1α Signaling, Aryl Hydrocarbon Receptor Signaling and Xenobiotic Metabolism Signaling pathways. The upregulated gene *S100G* particiapaptes in VDR/RXR Activation pathway. The downregulated gene *ADORA2B* plays a role in cAMP-mediated Signaling and G-Protein Coupled Receptor Signaling. The downregulated gene *S100G* contributes to TR/RXR Activation pathway. Through the analysis of Ingenuity Physiological System Development and Function, the above four genes: *ADORA2B*, *ARNT*, *DIO3* and *S100G* have been well studied and found to be involved in different physiological processes and the details were summerized in Table 3.

**Table 2:** Pathway analysis of 15 gene markers

Ingenuity Canonical Pathways	-Log(*P*-value)	Ratio	Molecules
Hypoxia Signaling in the Cardiovascular System	1.45E00	1.43E-02	ARNT
Renal Cell Carcinoma Signaling	1.41E00	1.37E-02	ARNT
VDR/RXR Activation	1.36E00	1.25E-02	S100G
TR/RXR Activation	1.33E00	1.06E-02	DIO3
VEGF Signaling	1.33E00	1.03E-02	ARNT
HIF1α Signaling	1.28E00	9.52E-03	ARNT
Aryl Hydrocarbon Receptor Signaling	1.12E00	6.37E-03	ARNT
cAMP mediated Signaling	1.07E00	6.1E-03	ORA2B
G-Protein Coupled Receptor Signaling	9.61E-01	4.59E-03	ORA2B
Xenobiotic Metabolism Signaling	8.56E-01	3.4E-03	ARNT

**Table 3:** Physiological functional anlaysis of 15 gene markers

Category	*P*-value	Molecules
Cardiovascular System Development and Function	5.75E-04-2.57E-02	ADORA2B, ARNT
Tissue Morphology	5.75E-04-1.15E-03	ADORA2B, ARNT
Embryonic Development	1.15E-03-5.17E-03	S100G, ARNT
Hematological System Development and Function	2.3E-03-2.3E-03	ADORA2B
Organ Morphology	2.3E-03-7.46E-03	ARNT
Reproductive System Development and Function	2.87E-03-3.9E-02	S100G, ARNT
Endocrine System Development and Function	3.45E-03-3.35E-02	DIO3, ARNT
Tissue Development	5.17E-03-5.17E-03	ARNT
Skeletal and Muscular System Development and Function	6.89E-03-6.89E-03	ADORA2B
Digestive System Development and Function	7.46E-03-7.46E-03	ARNT
Hepatic System Development and Function	7.46E-03-3.12E-02	ARNT
Organismal Survival	1.95E-02-1.95E-02	DIO3, ARNT
Organismal Development	3.4E-02-3.4E-02	DIO3
Organ Development	3.9E-02-3.9E-02	ARNT

### Identification of gene markers to separate individual metals

**Fig. 4:**
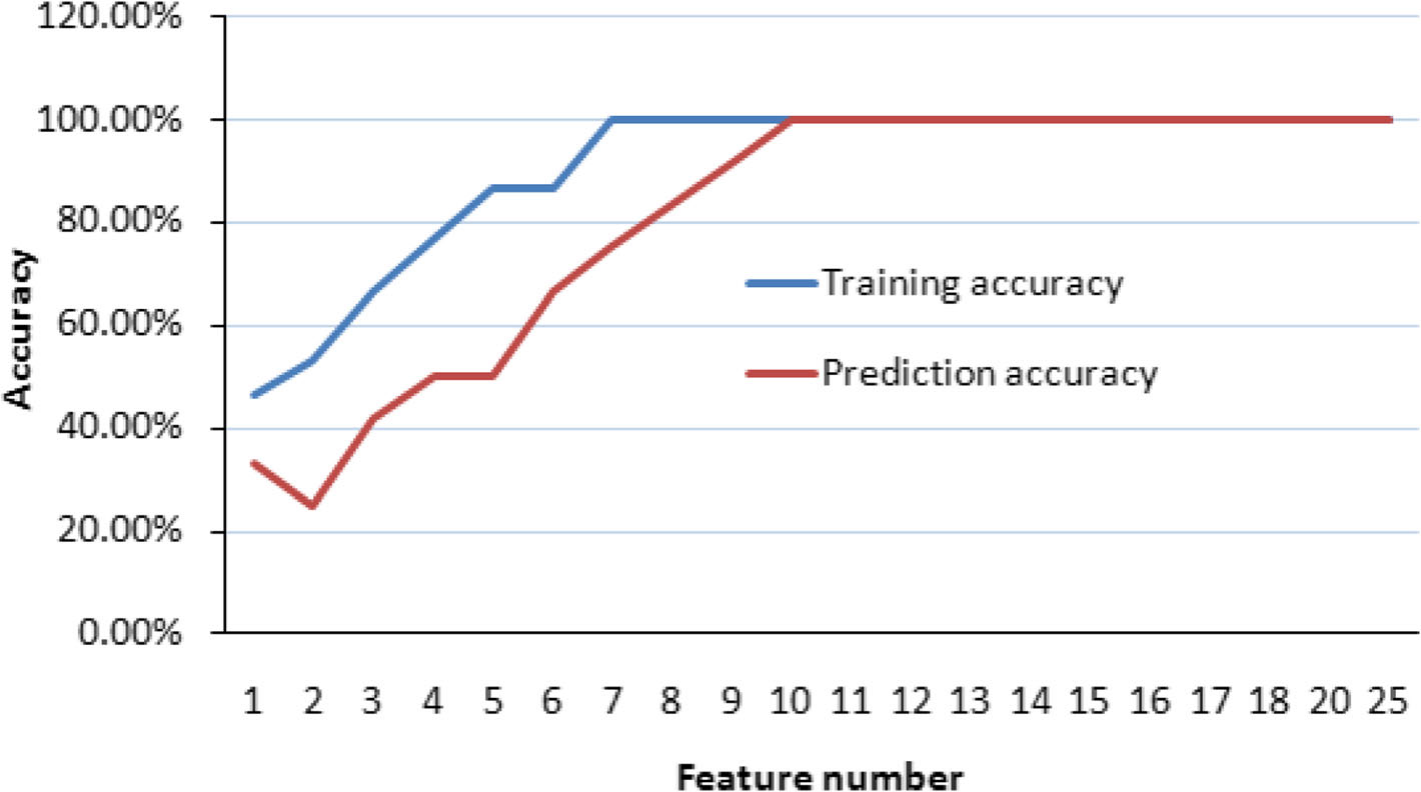
Identification of gene markers to separate individual metals. Both the training accuracy and prediction accuracy for 2007 dataset (Dataset 1) were increased with the the feature number increase. When the feature number was up to 7 and above, the training accuracy reached 100%

Since we could successfully discriminate metal from non-metal toxicants, whether individual metals could be separated from each other by gene expression profiles. For this purpose, we used the SVM-FRE feature selection method to train only metal samples in Dataset 2 to predict metal samples in Dataset 1 based on libSVM classification algorithm. The training accuracy and predicton accuracy were tested using the most 25 highly ranked features. As shown in Fig. 4, When the feature number was increased, both the training accuracy and prediction accuracy for Dataset 1 were increased. When the feature number was up to 7 and above, the training accuracy reached 100%. When the feaure number grew to 10 and above, the prediction accuracy also reached 100%, and there was no single sample that was mis-predicted. Therefore, it was comfirmed that using the 10 probe sets could accurately predict 9 individual metals.

### Gene expression pattern and functional analysis of the gene markers that distinguish individual metals

**Fig. 5:**
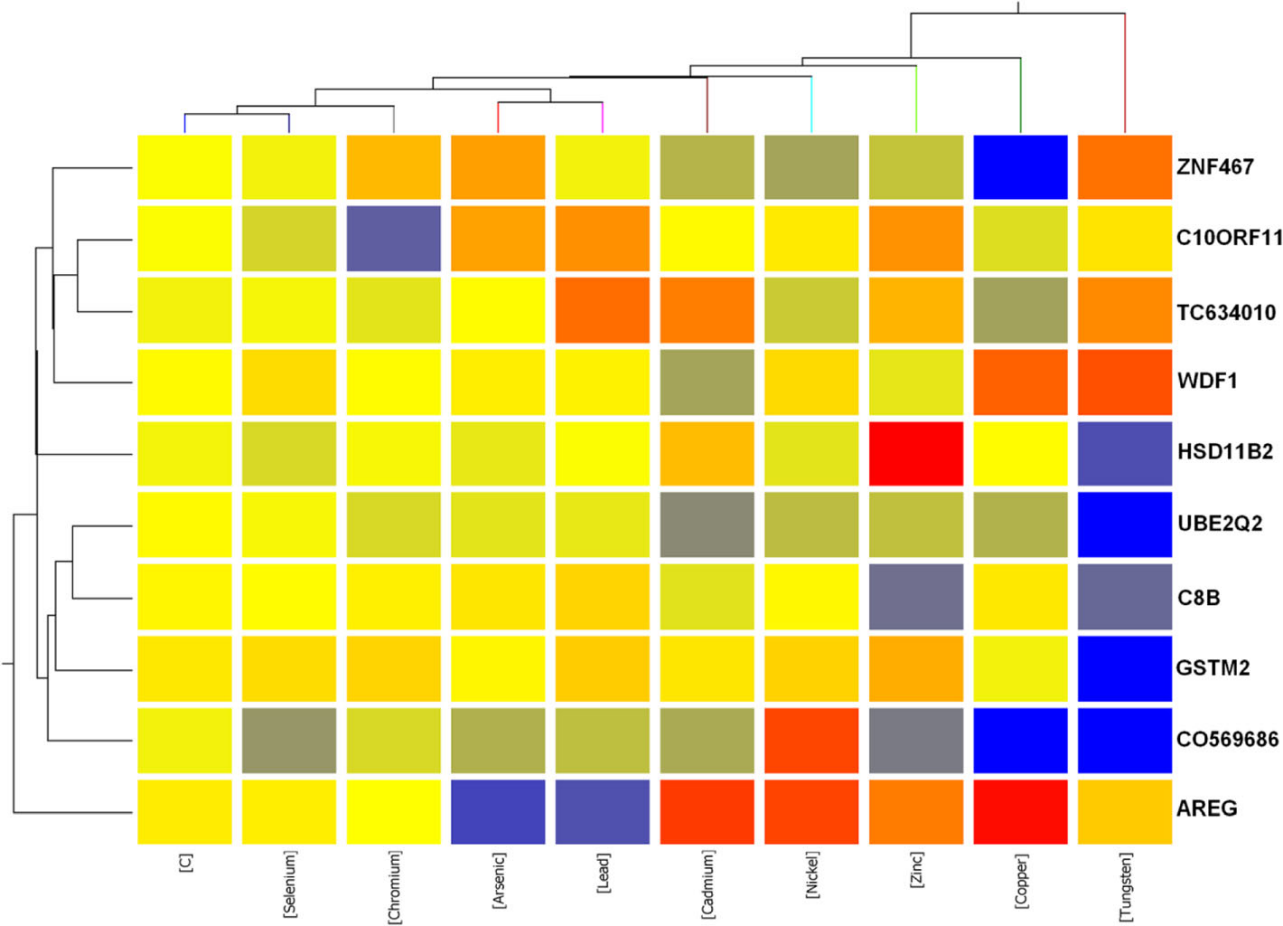
Heatmap shows the gene expression pattern and functional analysis of the gene markers that distinguish individual metals

To check how these two probe sets behaved in these 9 metals, we performed hierarchical clustering on both rows and columns across averaged metal and metal related control samples. As demonstrated in Fig. 5, the gene expression pattern of these 10 genes regulated by Tungsten was significantly different from other metals. Tungsten was the strongest regulator for the 10 gene markers. All these 10 markers’ expression was evidently affected by Tungsten. Compared to control, half genes were significanly upregulated and half genes were significantly downregulated by Tungsten.

Interestingly, it looked that we could find specific gene markers for almost all the metals. The gene zinc finger protein 467 (*ZNF467*) was downregaulted much more by Copper than any other metals. The gene chromosome 10 open reading frame 11(*C10ORF11*) was only significantly downregulated by Chromium but was up or marginally downregulated or remained no change by other metals. *TC6340110*’s expression was elevated the highest by Lead. WD40 and FYVE domain containing 1 (*WDF1*)‘s expression was repressed the highest by Cadmium. Hydroxysteroid (11-beta) dehydrogenase 2 (*HSD11B2*) was induced the strongest by Zinc. Glutathione S-transferase mu 2 (*GSTM2*) was only strongly downregulated by Tungsten. C0569686 was only significantly upregulated by Nickel. The gene amphiregulin (*AREG*) was strongly repressed by Arsenic and Lead, and Arsenic was stronger than Lead. Selenium had an overall weak regulation for these 10 gene markers, and it clustered in a subgroup with control. It was hard to find a specific marker for Selenium.

Well studied genes in the 10 gene marker list included *GSTM2*, *HSD11B*, *AREG*, *C8B* etc. Their functions are summarized in Tables 3 and 5. For instance, the gene *GSTM2* has been found to be involved in multiple pathways, such as Glutathione Metabolism, PXR/RXR Activation, Metabolism of Xenobiotics by Cytochrome P450, Aryl Hydrocarbon Receptor Signaling, NRF2-mediated Oxidative Stress Response, LPS/IL-1 Mediated Inhibition of RXR Function and Xenobiotic Metabolism Signaling pathways (Table 4). HSD11B participates in C21-Steroid Hormone Metabolism, and Androgen and Estrogen Metabolism pathways (Table 4), as well as in the physiological processes such as Connective Tissue Development and Function, Endocrine System Development and Function, Organ Morphology, Tissue Development, Skeletal and Muscular System Development and Function, Tissue Morphology, Cardiovascular System Development and Function and Nervous System Development and Function (Table 5).

**Table 4:** Pathway analysis of 15 gene markers

Ingenuity Canonical Pathways	-Log(*P*-value)	Ratio	Molecules
C21-Steroid Hormone Metabolism	2.07E00	1.41E-02	HSD11B2
Complement System	1.84E00	2.78E-02	C8B
Glutathione Metabolism	1.65E00	1.02E-02	GSTM2
PXR/RXR Activation	1.54E00	1.16E-02	GSTM2
Androgen and Estrogen Metabolism	1.51E00	6.99E-03	HSD11B2
Neuregulin Signaling	1.43E00	1E-02	AREG
Metabolism of Xenobiotics by Cytochrome P450	1.27E00	4.76E-03	GSTM2
Aryl Hydrocarbon Receptor Signaling	1.24E00	6.37E-03	GSTM2
NRF2-mediated Oxidative Stress Response	1.14E00	5.41E-03	GSTM2
LPS/IL-1 Mediated Inhibition of RXR Function	1.1E00	4.88E-03	GSTM2
Xenobiotic Metabolism Signaling	9.73E-01	3.4E-03	GSTM2

**Table 5:** Physiological functional anlaysis of 15 gene markers

Category	*P*-value	Molecules
Connective Tissue Development and Function	4.32E-04-3.53E-02	HSD11B2, AREG
Endocrine System Development and Function	4.32E-04-4.31E-03	HSD11B2
Organ Morphology	4.32E-04-2.59E-03	HSD11B2, AREG
Reproductive System Development and Function	4.32E-04-1.8E-02	AREG
Tissue Development	4.32E-04-3.53E-02	HSD11B2, AREG
Organ Development	8.63E-04-7.74E-03	AREG
Skeletal and Muscular System Development and Function	1.29E-03-3.53E-02	HSD11B2, AREG
Tissue Morphology	1.29E-03-4.74E-03	HSD11B2, AREG
Embryonic Development	3.02E-03-3.02E-03	AREG
Tumor Morphology	2.69E-02-4.61E-02	AREG
Cardiovascular System Development and Function	2.73E-02-2.73E-02	HSD11B2
Nervous System Development and Function	2.86E-02-4.41E-02	HSD11B2, AREG
Hair and Skin Development and Function	2.98E-02-2.98E-02	AREG

## Discussion

Previous study has used toxic genomics strategies to predict the toxicity of various compounds based on possible similar toxicity and potential molecular mechanisms of various chemicals [17]. For example, gene expression profiling has been successfully applied to the classification of rodent poisons and to distinguish between hepatotoxic and non-hepatotoxic compounds. Similarly, transcriptional profiles have also been used to predict environmental safety and industrial chemicals associated with cancer [18]. In this study, we established models to predict non-metal and metal contamination by identifying gene markers based on gene expression profiles.

In vitro cultured cells are widely used because of their clear background, consistent source, easy culture, easy control of culture conditions, and good reproducibility. Chip technology can quickly and efficiently screen differential genes. Non-metal and metal compounds can cause different gene expression profiles in cells. In this study, we spent2 years to create two datasets, as two models, using probes to generate three feature sets that are mutually validated.

We applied two selection methods: SVM-RFE and InfoGain, and then used libSVM’s classification algorithm to build predictive models: metal and non-metal. Cross-validation to assess their accuracy is quite high, indicating that gene expression profiles can distinguish metals from non-metallic poisons.

By cross validating the sample classification of the corrected data sets, we found that based on the 15 probe sets, the cross-validation error for Dataset 2 and the joint dataset is 0. The prediction error of Dataset 1 is also 0. Therefore, these 15 probe sets can be a good signature to descriminate metal from non-metal toxicants.

In this study, we analyzed the expression profiles of metal and non-metal genes. The metal gene expression profile has 8 genes belonging to the membrane, indicating the metal is mainly targeted to membrane proteins which is different from non-metals. Then, we analyzed each gene expression profile of a metal, in addition to Selenium, specific gene markers were identified for another 8 metals. Based on the libSVM classification algorithm, the SVM-FRE feature selection method was chose to separate individual metal, and this method has been confirmed with better performance than other method [8, 15, 16].

Based on the classification system and gene expression profiling, these 10 genes have potential applicability in predicting what class a new compound belongs to when a gene expression profile is available with the probe sets. In general, genes selected as biomarkers show similar expression patterns [19]. The gene expression pattern of these 10 genes regulated by Tungsten was significantly different from other metals. All these 10 genes’ expression was evidently affected by Tungsten, which significantly unregulated half of the genes and significantly down regulated the other half. Recently years, emerging studies show that tungsten is an environment toxicant, not only alone but also in combination; however, its potential risk of exposure on human is still unclear [20]. So, our findings support further investigation into the toxicities of tungsten and its potential molecular mechanism.

Our experiments results indicated that the 10 genes could be used to accurately predict 9 individual metals. Moreover, we identified some pathways were involved in the association with the exposures to toxic metals. However, the precise role of these genes in most pathways is still unclear and warrants further investigation, our findings also need to be verified by other well-designed cohort studies.

## Conclusions

This study demonstrates that using a microarray classifier analysis, not only can create diagnostic classifiers for predicting an exact metal contaminant from a large scale of contaminant pool with high prediction accuracy, but also can identify valuable biomarkers to help understand the common and underlying toxic mechanisms induced by metals. Our findings highlight a potential utility of gene markers of toxic metals for public health assessment, prevention, and precision health in the future.

## Data Availability

Not applicable.

## Abbreviations

SVM-RFE: Support vector machine - recursive feature elimination
LibSVM: Library for support vector machines
DMSO: Dimethyl sulfoxide
ADORA2B: Adenosine A2b receptor
FAM70B: Family with sequence similarity 70, member B
FAM174B: family with sequence similarity 174, member B
KHDRBS3: KH domain containing, RNA binding, signal transduction associated 3
DIO3: Type 3 iodothyronine deiodinase
SLC1A5: Solute carrier family 1 (neutral amino acid transporter) member 5
RTN2: Reticulon 2
CDIG2: Cadmium-inducible gene
FAM12B: Family with sequence similarity 12, member B (epididymal)
ARNT: Aryl hydrocarbon receptor nuclear translocator
IGH-6: Immunoglobulin heavy chain 6 (heavy chain of IgM)
AHNAK2: AHNAK nucleoprotein 2
S100G: S100 calcium binding protein G
